# Extensive-Stage Small Cell Carcinoma Transformation From EGFR Del19-Mutant Lung Adenocarcinoma on Gefitinib at the Twelfth-Year Follow-Up Case Report

**DOI:** 10.3389/fonc.2021.564799

**Published:** 2021-03-18

**Authors:** Victor C. Kok, Chien-Kuan Lee, Yu-Hsin Chiang, Ming-Chih Wang, Yen-Te Lu, Chiu-Chun Cherng, Pei-Yu Lee, Ke-Bin Wang

**Affiliations:** ^1^ Division of Medical Oncology, KTGH Cancer Center, Kuang Tien General Hospital, Taichung, Taiwan; ^2^ Disease Informatics Research Group, Asia University Taiwan, Taichung, Taiwan; ^3^ Department of Pathology, Kuang Tien General Hospital, Taichung, Taiwan; ^4^ Division of Chest Surgery, Department of Surgery, Kuang Tien General Hospital, Taichung, Taiwan; ^5^ Department of Radiation Oncology, Kuang Tien General Hospital, Taichung, Taiwan; ^6^ Division of Neurosurgery, Department of Surgery, Kuang Tien General Hospital, Taichung, Taiwan; ^7^ Department of Diagnostic and Intervention Radiology, Kuang Tien General Hospital, Taichung, Taiwan; ^8^ Department of Nuclear Medicine, Kuang Tien General Hospital, Taichung, Taiwan

**Keywords:** small cell carcinoma transformation, EGFR-mutant lung adenocarcinoma, Del-19 mutation, topotecan (PubChem CID: 60700), case report

## Abstract

**Introduction:**

The acquired resistance mechanisms in patients with epidermal growth factor receptor (EGFR)-mutant lung cancer, particularly adenocarcinoma (ADC), following treatment with an EGFR tyrosine kinase inhibitor (TKI) have received extensive investigations. The phenotypic transformation to small cell carcinoma (SCCT) has been estimated to occur in approximately 3 to 10% of patients treated with an EGFR-TKI. The prognosis after SCCT is extremely poor.

**Case Study:**

We report about SCCT that occurred 45 months after the initial diagnosis of ADC in an East Asian never-smoker woman with advanced-stage *EGFR* Del-19-mutant lung ADC treated with combined chemoradiotherapy before the era of insurance coverage for EGFR-TKIs in this country and subsequently gefitinib; deletion at codon 746–750 in exon 19 of the *EGFR* gene was ascertained in the original formalin-fixed paraffin-embedded lung biopsy tissue. Spinal cord compression at thoracic-12 level from SCCT was successfully relieved with neurosurgical treatment, chemotherapy with etoposide and cisplatin, and radiotherapy, while gefitinib treatment was maintained. Eleven months later, SCCT relapsed in the lung parenchyma, which was resected and was found to be sensitive to second-line weekly topotecan. Prophylactic cranial irradiation was subsequently administered. SCCT was confirmed by MALDI-TOF MS analysis of formalin-fixed paraffin-embedded tissues demonstrating the same exon 19 deletion. At the 12th-year follow-up, the patient remains relapse free with very good performance status. The novelty of this case is the successful interdisciplinary team effort to correct the spinal cord compression by maintaining the patient in an ambulatory state, non-stop use of gefitinib justified by the presence of activating EGFR mutation in SCCT tumor cells, and aggressive dose-intensive chemotherapy and radiotherapy for the SCCT that leads to an unprecedented prolonged remission and survival. This case also supports the observation that SCCT is chemotherapy sensitive, and thus, re-biopsy or complete tumor excision is recommended to understand the mutation profiles of the current tumor. Aggressive prudent administration of systemic chemotherapy obtaining optimal dose intensity leads to the successful management of the patient.

## Introduction

According to the literature, small cell carcinoma transformation (SCCT) from epidermal growth factor receptor (EGFR)-mutant adenocarcinoma (ADC) of the lung occurs at a rate of 3–10% (1). The prognosis of SCCT is very poor, with just one in 67 patients (1.5%) surviving to 80 months (1). For patients already in the extensive stage at the time of transformation, the prognosis is even worse because the administration of remissive treatment for SCCT is often less dose-intensive owing to several reasons such as clinical deterioration, organ dysfunctions, or pessimistic views held by the treatment team and the patient. Here, we report about a patient with histopathological and molecular genetic confirmation of SCCT in the extensive stage who holds a record-long durable remission and reasonably good performance status and quality of life.

## Case Description

**Figure 1:**
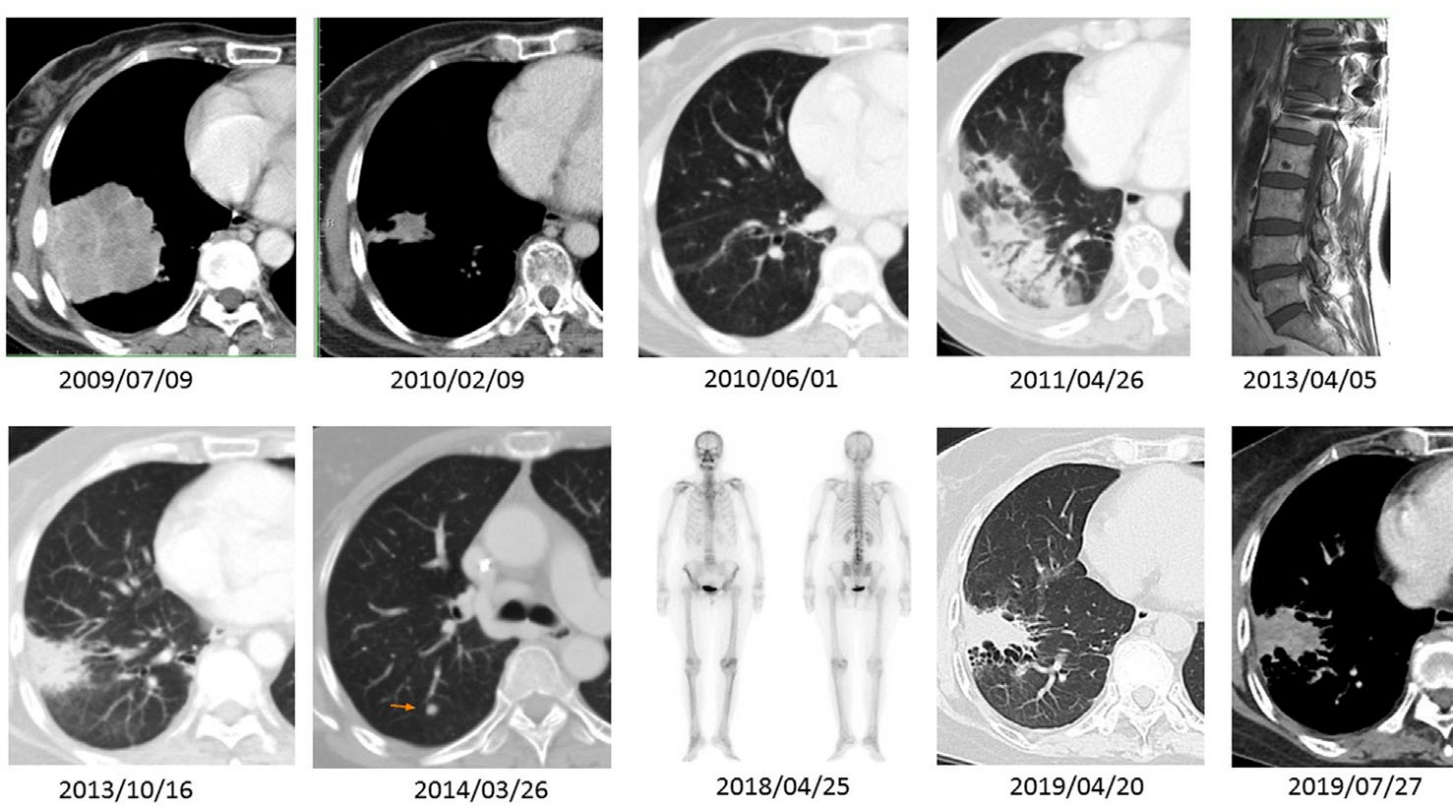
A series of imaging studies shown here are CT scans of the chest in either soft tissue or lung windows, a magnetic resonance imaging (MRI) of the spine, and a bone scan obtained spanning 10 years period. The patient had an EGFR Del-19-mutant ADC (2009/07/09). The ADC showed excellent tumor regression after five cycles of induction chemotherapy and three months of gefitinib (2010/02/09 and 2010/06/01). During effective gefitinib treatment, the patient developed an uneventful transitory pneumonitis documented on 2011/04/26. She received the same treatment until 2013/04/05, when she developed extensive-stage SCCT, causing impending spinal cord compression. There was a relapse of small cell carcinoma in the lung parenchyma (2014/03/26) after combined chemoradiotherapy with etoposide–cisplatin and prophylactic cranial irradiation. However, after lung tumor resection and second-line weekly topotecan treatment, the SCCT went into complete remission. Follow-up scans (2018/04/25 through 2019/07/27) demonstrate a quiescent bone scan and fibrotic changes at the previous ADC site.

**Figure 2:**
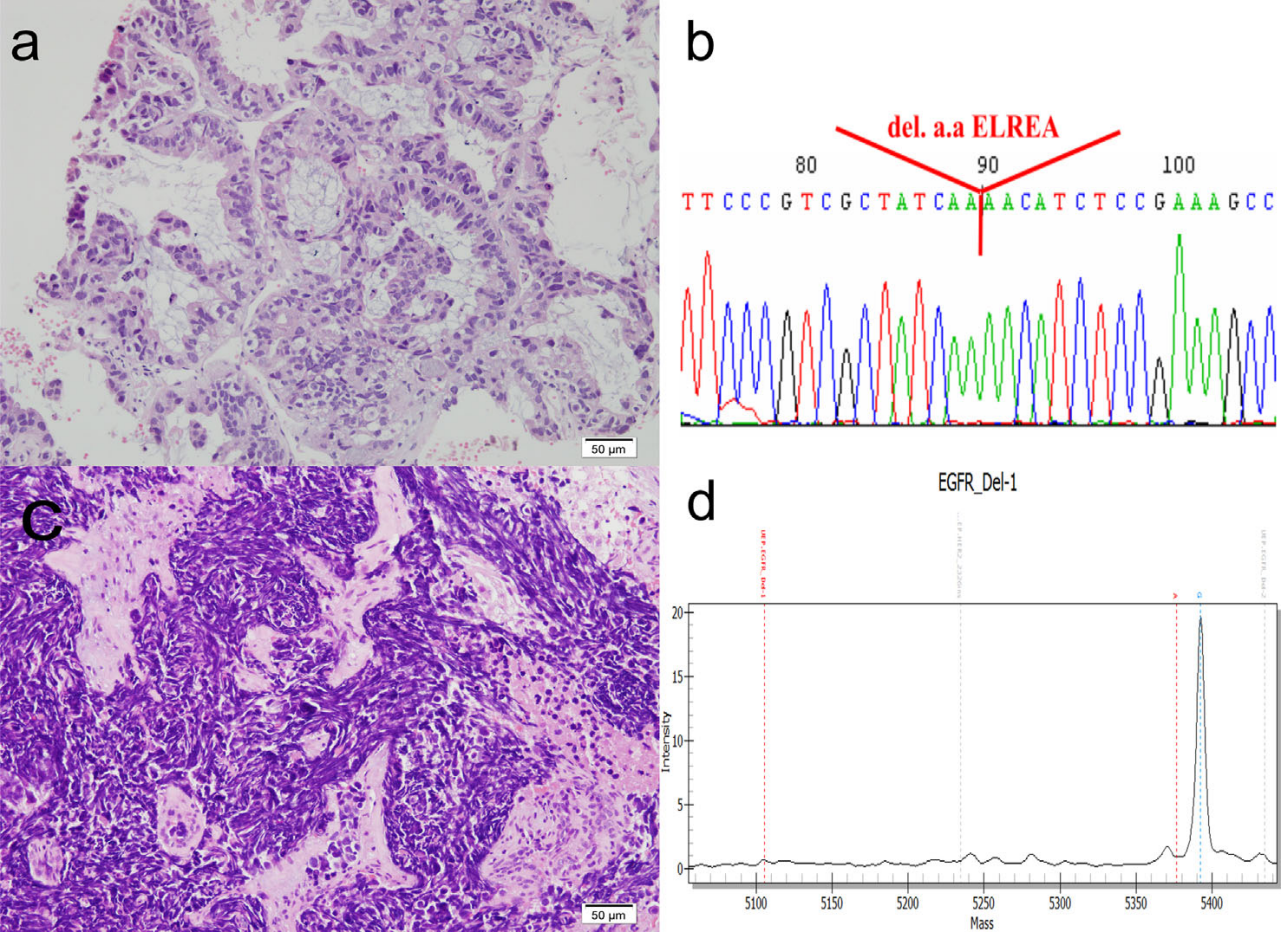
Histomorphology and mutation analysis of the *EGFR* gene. **(A)** The original lung biopsy showing ADC arranged in an acinar architecture. **(B)** Deletion at codon 746–750 in exon 19 of the *EGFR* gene in the original lung biopsy. The DNA, extracted from paraffin sections, was subjected to PCR amplification for exons 18, 19, 20, and 21 of the *EGFR* gene. These amplicons were then sequenced. The result shows codon 756–750 deletion in exon 19, resulting in deletion amino acids ELREA. **(C)** Repeat lung biopsy revealing the transformation of small cell carcinoma, which is demonstrated by sheets of crushed, small sized tumor cells, with high nuclear to cytoplasmic ratio, smudged chromatin and minimal cytoplasm. **(D)** MALDI-TOF MS of repeat lung biopsy indicating that the deletion in codon 746–750 in exon 19 of the *EGFR* gene was retained. The quality and quantity of extracted DNAs (concentration: 4.85 ng/ul) are measured by NanoDrop (ND-1000), and the mutation detection is measured by the MassARRAY genotyping (SEQUENOM) incorporating a MALDI-TOF mass spectrometer. Result confirmed the presence of Type 1 Exon 19 deletion: c.2235_2249del p (Glu746_Ala750del). **(D)** shows the spectrogram, mass (X-axis) versus intensity (Y-axis), and the diagnostic mass peaks from 5,354 to 5,400.

A 65-year-old never-smoker woman weighing 62 kg presented to our hospital in July 2009 with persistent severe mid-back pain. Bone scan on July 6, 2009 showed increased ^99m^Tc-labeled methylene diphosphonate uptake at the thoracic 12 level and the third right rib, in addition to the hypertrophic pulmonary osteoarthropathy (HPOA) of bilateral femurs and tibias, which were compatible with bone metastases. Chest computed tomography (CT) revealed a huge lobulated lung mass measuring up to 6.87 cm in the right lower lobe, with a daughter nodule in the same lung lobe (**Figure 1**, upper panel, leftmost). Right pleural malignant effusion was apparent. The original lung biopsy showed atypical glands with lepidic and acinar architecture and enlarged and hyperchromatic nuclei (**Figure 2A**). A diagnosis of moderately differentiated pulmonary ADC, cT4 N2 M1b Stage IV, was made. *EGFR* analysis performed using targeted exon sequencing identified the deletion of codon 746–750 in exon 19, resulting in the deletion of the amino acids ELREA (**Figure 2B**). The first-line use of gefitinib was not authorized under the national health insurance program in this country until June 2011, whereas second-line gefitinib after failing prior chemotherapy was allowed since November 2007. From July 20 until September 21, 2009, three cycles of full-dose paclitaxel + cisplatin were administered and then discontinued because of grade III sensory neuropathy by the Common Terminology Criteria for Adverse Events (CTCAE). For the 4th cycle of chemotherapy, the regimen was switched to gemcitabine + cisplatin. Follow-up chest CT showed partial remission. Three cycles of paclitaxel + cisplatin and two cycles of gemcitabine + cisplatin were administered. Gradual improvement of the sensory neuropathy down to CTCAE grade I was noted. The patient then started receiving gefitinib as second-line therapy on November 2, 2009, a treatment that currently ongoing. With persistent disease despite a partial remission, we prescribed gefitinib for tumor control. In addition, from October 19, 2009 to September 5, 2011, up to 25 cycles of 4-weekly pamidronate were administered aiming to mitigate the risk of any skeletal events. When bone metastases were stabilized, we delivered a course of radiotherapy with radical intent for the lung tumor as for locally advanced lung cancer. From December 29, 2010 to February 16, 2011, a course of palliative radiotherapy with intensity modulated radiotherapy (IMRT) (66 Gy/33 fractions/50 days) was administered to the RLL lung tumor. Chest CT on July 7, 2012 revealed complete regression of the lung ADC with fibrotic changes.

However, the patient developed bilateral leg weakness resulting from spinal cord compression in April 2013 (**Figure 1**, upper series, rightmost panel). She underwent emergent laminectomy of thoracic-11-lumbar-1 and curettage of thoracic-12 vertebra on April 15, 2013. Lung and metastatic bone lesion biopsies revealed small tumor cells forming a sheet-like growth pattern with scant cytoplasm, finely granular chromatin, nuclear molding, necrosis, and frequent mitosis. Immunohistochemical staining showed positivity for neuroendocrine markers, including synaptophysin, chromogranin A, and CD56. The original primary lung biopsy was retrieved for retrospective review and showed only adenocarcinoma with no evidence of combined histologies. Two additional pathologists independently confirmed the final pathological diagnosis of SCCT (**Figure 2C**). A course of IMRT with 30 Gy/10 fractions/13 days was administered to the T-11 to L1 spinal metastases from April 25 to May 7, 2013. From May 6 to July 11, 2013, four cycles of etoposide–cisplatin regimens were completed. Complete remission occurred with no tumor activity, normal lactate dehydrogenase level, and improving performance status. Prophylactic cranial irradiation was performed with IMRT (25 Gy/10 fractions/12 days) from August 12 to August 23, 2013.

**Figure 3:**
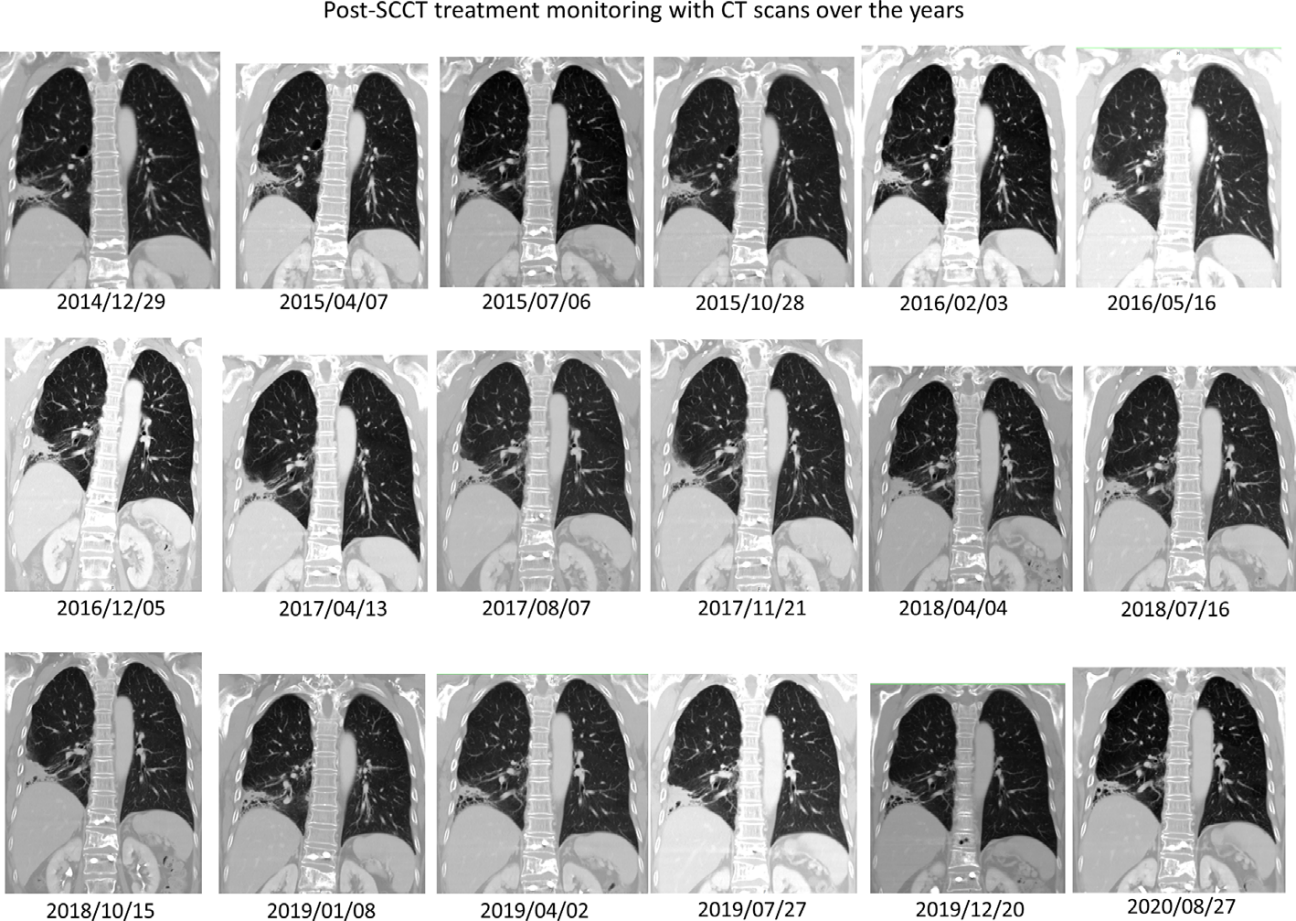
Post-SCCT induction treatment monitoring and long-term follow-up with CT scans over the years. The schema diagram illustrates a series of computerized tomography coronal sections through the right lower lobe primary adenocarcinoma, which has turned into post-treatment fibrosis and recently asymptomatic bronchiectatic change. Through these non-progressive CT scans, one can be confident post-hoc that a complete response was attained in 2014 after complete treatment for SCCT. There is no evidence of SCCT recurrence over time.

On March 26, 2014, a subcentimeter nodule was noted in the right upper lung lobe (**Figure 1**, lower panel, second left). Thoracoscopic wedge resection was performed, which revealed SCLC relapse. We considered recurrent cancer as a failure to the first-line etoposide–cisplatin; hence, we prescribed topotecan as the second-line treatment for recurrent SCLC. From April 21 to August 8, 2014, six weekly cycles of single-agent topotecan at 4 mg/m^2^ were administered. EGFR mutation analysis using matrix-assisted-laser-desorption-ionization time-of-flight mass spectrometry (MALDI-TOF MS) for SCLC identified the same deletion in exon 19 (**Figure 2D**). The patient continued to receive treatment with gefitinib. The justification for continuing gefitinib was the presence of the activating EGFR mutation in the transformed histology cancer. On August 8, 2018, the patient met with a traffic accident that resulted in a right pelvic bone fracture and left distal radial bone fracture. She developed lumbar-3 benign compression fracture + L4-5 spinal stenosis, for which L3 vertebroplasty and L4–5 decompression laminectomy were performed on April 30, 2018. She recovered nicely thereafter. The patient currently to receive treatment with gefitinib was with tolerable adverse reactions such as mild intermittent diarrhea (CTCAE grade I), acne-form rash (grade I), and paronychia (grade I). We assessed the evolution of the HPOA through serial images of whole-body bone scans using technetium-99m with methylene diphosphonate, which were performed on July 7, 2009, March 5, 2013, April 2, 2018, and July 18, 2018, respectively. The increased methylene diphosphonate uptake at the bilateral femurs and tibias was compatible with the HPOA, also known as Pierre Marie–Bamberger syndrome (2). The serial whole-body bone scans demonstrated stabilized disease with neither cortical destruction nor pathological fractures over the years. As compared with the initial scan images, there was no new lesion during follow-up. **Figure 3** illustrates a series of CT scans at regular follow-ups after the induction treatment for SCCT over the years. As of February 22, 2021, she has an excellent performance status and can take care of her grandchildren at home.

## Discussion

We performed a retrospective analysis of the primary tumor to see if it could have been a combined adenocarcinoma with *de novo* small cell carcinoma at the outset. The original biopsy had been reviewed and showed only adenocarcinoma. Two additional pathologists independently confirmed the final pathological diagnosis. However, to completely exclude a *de novo* small cell carcinoma component, it would require complete resection of the primary tumor, which was not performed considering that patient’s clinical status which was not suitable for operation. There was a slim chance, if any, that the original tumor might be a combined adenocarcinoma and *de novo* small cell carcinoma.

There are several distinct mechanisms of acquired resistance to EGFR inhibitors, including the acquisition of an additional somatic mutation in *EGFR* (T790M mutation) and amplification of the *MET* proto-oncogene or the *ERBB2* proto-oncogene (3, 4). Histological transformation of *EGFR*-mutant ADC to SCLC is another mechanism of acquired resistance (3, 5). In the first described case that was treated with erlotinib, repeat biopsy showed SCLC with the original *EGFR* exon 19 deletion (6). In several reported case series, the diagnosis of SCLC in repeat biopsies was confirmed based on morphology and immunohistochemical staining for synaptophysin, chromogranin, or NCAM (5). Mutation analysis of *EGFR* from both the original and repeat biopsy specimens at the time of resistance showed that SCCT tumor samples retained their original *EGFR*-activating mutation (3), suggesting that the transformed tumor phenotype is a mechanism of resistance to treatment.

The transformation from ADC to SCLC can occur independently of the *EGFR* mutational status and is not exclusively the result of EGFR inhibition (7). Current data suggest that SCCT is more common in *EGFR*-mutant ADC treated with EGFR inhibitors than in wild-type ADC. However, this observation could be biased because biopsy at progression in NSCLC is not generally feasible and a greater number of repeat biopsy samples are performed in patients with *EGFR*-mutant cancers than in those with non-*EGFR*-mutant ADC. Larger studies will be needed to identify which subsets of NSCLC are the most prone to SCCT.

The uniqueness of this case report rests upon the MALDI-TOF MS confirmation of the same *EGFR* Del-19 mutation in SCCT; aggressive combined modality chemoradiotherapy, including PCI; and continuation of gefitinib treatment to control susceptible cancer cells. Without concrete evidence to guide management and some anecdotal reports had observed that gefitinib could be useful in EGFR-mutant small cell carcinoma (8–10), we adopted the strategy of gefitinib maintenance for the patient. Nevertheless, this case report’s unique outcome cannot justify the necessity of maintaining gefitinib in all the patients with transformed carcinoma harboring an activating EGFR mutation. Suppose we could be sure of the EGFR-mutant residual disease status and the EGFR TKI-refractoriness after combined chemoirradiation, can we only be able to decide the timing of gefitinib discontinuation. For example, it is tempting to think that perhaps a series of high-sensitivity liquid biopsies to detect EGFR mutation in peripheral circulation might guide the appropriate timing of discontinuation. This case also strongly supports evidence that SCCT is chemotherapy sensitive even after relapse from the first-line chemotherapy. Would EGFR mutation associate with chemosensitivity in transformed small cell carcinoma? Two retrospective cohort studies (11, 12) suggested that EGFR mutation may predict a greater likelihood of response to cytotoxic chemotherapy in NSCLC patients receiving chemotherapy as a front-line treatment. Whether EGFR mutation also predicts greater cytotoxic chemosensitivity in transformed small cell carcinoma is a valid research question to pursue.

For classical small cell lung cancer having no EGFR mutation, the current standard systemic regimen is immunochemotherapy targeting the programmed cell death protein 1 (PD-1)/PD-ligand 1 (PD-L1) pathway with efficacy as demonstrated in two large prospective randomized controlled trials (13–15). It could be another story for non-classical EGFR-mutant transformed small cell carcinoma since some research data do not support the use of PD-1/PD-L1 immune checkpoint inhibitors for SCCT owing to the lack of efficacy observed in retrospective series (1, 16).

In this case report, MALDI-TOF MS was performed to diagnose the EGFR mutation in SCCT. The turn-around time for MALDI-TOF MS to deliver a quick diagnosis of EGFR mutation is as short as 4 days, and when compared with Sanger sequencing, both analytical sensitivity and specificity are 100% (17).

In East Asia such as Taiwan, the prevalence of *EGFR* Del-19 mutation in lung cancer is approximately 23.1% (17). Patients with Del-19 mutation respond very well to EGFR-TKI. Interestingly, 70–93% of SCCT harbor the Del-19 mutation (1, 18). Therefore, we propose that whenever acquired resistance develops during EGFR-TKI treatment in patients with the Del-19 mutation, re-biopsy should be indicated because SCCT can be swiftly discovered and chemotherapy can be administered sooner.

The molecular mechanisms underlying SCCT are beginning to be understood. A study by researchers of the Massachusetts General Hospital identified that every case of SCCT has loss of the tumor suppressor *RB* before transformation (19). The nature of the universal genetic loss of *RB1* implies that this may be a necessary event for SCCT to emerge (19, 20). Hence, theoretically, if this *RB* loss can be detected by liquid biopsy during patient treatment monitoring, we may be able to start appropriate treatment early after transformation.

Implementation of precision oncology improves patients’ prospects after being diagnosed with EGFR-mutant ADC and even in the stage of the rare occurrence of histologic SCCT. Appropriate dose intensity salvage chemotherapy, prophylactic cranial irradiation, plus good dose radiotherapy should always be the number one option for fit patients. This case report lends strong support to the practice that should always consider rendering the most durable remission as the treatment goal while treating a patient with EGFR-mutant lung cancer having SCCT as the acquired resistance from EGFR-TKI therapy.

## Patient Perspective

‘It was disastrous to know that I got lung cancer in so late the stage. I trust my doctor and whose medical team from the very beginning, and I receive all the treatment plans they devise for me. I don’t think I can understand why I needed to receive those complex treatments, although the doctor has told me from time to time. I never thought that I could accompany and watch my grandson grow up for the past twelve years. I feel very happy to be able to take care of him personally. Now I can walk and prepare food for the family and perform other necessary activities of daily living. The remaining tingling sensation in my fingers and toes is negligible most of the time.’

## Ethics Statement

The patient provided written informed consent for academic publishing of her case.

## Author Contributions

All the authors took part in taking care of the patient. VCK and CKL drafted the manuscript. VCK prepared **Figures 1** and **3**. C-KL made **Figure 2**. All authors contributed to the article and approved the submitted version.

## Conflict of Interest

The authors declare that the research was conducted in the absence of any commercial or financial relationships that could be construed as a potential conflict of interest.
